# Retraction: The protective effect of propofol on ionizing radiation-induced hematopoietic system damage in mice

**DOI:** 10.1039/d4ra90144d

**Published:** 2024-12-05

**Authors:** Xiaoliang Han, Fengtao Sun, Ying Zhang, Jinyan Wang, Qingguo Liu, Ping Gao, Shubo Zhang

**Affiliations:** a Affiliated Hospital, North China University of Science and Technology Tangshan Hebei 063000 China mayastarfx2008@163.com; b Tangshan Gongren Hospital Tangshan Hebei 063000 China

## Abstract

Retraction of ‘The protective effect of propofol on ionizing radiation-induced hematopoietic system damage in mice’ by Xiaoliang Han *et al.*, *RSC Adv.*, 2019, **9**, 36366–36373, https://doi.org/10.1039/C9RA07262D.

The authors hereby wholly retract this *RSC Advances* article as the data from Fig. 1a, Fig. 2, Fig. 3a and Fig. 4d and 4e were first published by Xiaoliang Han *et al.* in ref. 1 and were reproduced in this paper without permission.

Signed: Xiaoliang Han, Fengtao Sun, Ying Zhang, Jinyan Wang, Qingguo Liu, Ping Gao and Shubo Zhang

Date: 27th November 2024

Retraction endorsed by Laura Fisher, Executive Editor, *RSC Advances*
